# High-Fat Diet: Bacteria Interactions Promote Intestinal Inflammation Which Precedes and Correlates with Obesity and Insulin Resistance in Mouse

**DOI:** 10.1371/journal.pone.0012191

**Published:** 2010-08-16

**Authors:** Shengli Ding, Michael M. Chi, Brooks P. Scull, Rachael Rigby, Nicole M. J. Schwerbrock, Scott Magness, Christian Jobin, Pauline K. Lund

**Affiliations:** 1 Department of Cell & Molecular Physiology and Department of Nutrition, University of North Carolina at Chapel Hill, Chapel Hill, North Carolina, United States of America; 2 Department of Gastroenterology, Vanderbilt University Medical Center, Nashville, Tennessee, United States of America; 3 Division of Biomedical and Life Sciences, School of Health and Medicine, Lancaster University, Lancaster, United Kingdom; 4 Division of Gastroenterology and Hepatology, Department of Medicine, University of North Carolina at Chapel Hill, Chapel Hill, North Carolina, United States of America; Sapienza University of Rome, Italy

## Abstract

**Background:**

Obesity induced by high fat (HF) diet is associated with inflammation which contributes to development of insulin resistance. Most prior studies have focused on adipose tissue as the source of obesity-associated inflammation. Increasing evidence links intestinal bacteria to development of diet-induced obesity (DIO). This study tested the hypothesis that HF western diet and gut bacteria interact to promote intestinal inflammation, which contributes to the progression of obesity and insulin resistance.

**Methodology/Principal Findings:**

Conventionally raised specific-pathogen free (CONV) and germ-free (GF) mice were given HF or low fat (LF) diet for 2–16 weeks. Body weight and adiposity were measured. Intestinal inflammation was assessed by evaluation of TNF-α mRNA and activation of a NF-κB^EGFP^ reporter gene. In CONV but not GF mice, HF diet induced increases in body weight and adiposity. HF diet induced ileal TNF-α mRNA in CONV but not GF mice and this increase preceded obesity and strongly and significantly correlated with diet induced weight gain, adiposity, plasma insulin and glucose. In CONV mice HF diet also resulted in activation of NF-κB^EGFP^ in epithelial cells, immune cells and endothelial cells of small intestine. Further experiments demonstrated that fecal slurries from CONV mice fed HF diet are sufficient to activate NF-κB^EGFP^ in GF NF-κB^EGFP^ mice.

**Conclusions/Significance:**

Bacteria and HF diet interact to promote proinflammatory changes in the small intestine, which precede weight gain and obesity and show strong and significant associations with progression of obesity and development of insulin resistance. To our knowledge, this is the first evidence that intestinal inflammation is an early consequence of HF diet which may contribute to obesity and associated insulin resistance. Interventions which limit intestinal inflammation induced by HF diet and bacteria may protect against obesity and insulin resistance.

## Introduction

It is now widely accepted that obesity is associated with low-grade chronic inflammation and that inflammation contributes to risk of insulin resistance and type 2 diabetes as well as other detrimental health consequences linked to obesity [1], [2], [3]. Most prior studies have focused on adipocytes as sources of inflammatory mediators in obesity. Circulating and adipose derived cytokines such as tumor necrosis factor-α (TNF-α) or interleukin-6 (IL-6) have been shown to be elevated in obese humans [4], [5], [6], [7] and this can be reversed with weight loss [8]. In animal models, multiple studies have demonstrated that diet-induced obesity is associated with increased expression of a number of proinflammatory cytokines or biomarkers of inflammation in adipose tissue [9], [10]. The mechanisms underlying obesity-associated inflammation are not fully defined. A number of studies support a concept that inflammation may derive from the accumulation of activated macrophages within adipose tissue and, particularly, surrounding enlarged adipocytes of obese animals or humans [10], [11]. The source of adipocyte-derived macrophages in obesity is not known but HF diet induces expression of adhesion molecules in adipose tissue, which are associated with leukocyte migration and adherence [9]. The gastrointestinal (GI) tract is another potential source of inflammation associated with diet or obesity that has not been extensively explored. Recent studies suggest that normal non-pathogenic enteric bacteria play a key role in diet-induced adiposity because GF mice were reported to have less body fat [12] and do not become obese or insulin resistant when subject to a HF diet [13]. Obesity is also associated with altered gut microbiota [14]. Gut bacteria metabolize indigestible polysaccharides to generate short-chain fatty acids and monosaccharides and promote their absorption and storage as fat [12]. In addition, gut bacteria increase vascularization and blood flow within the mucosa, facilitating nutrient absorption. Gut bacteria also suppress the expression and release of fasting induced adipose factor (Fiaf) from small intestine, resulting in increased activity of lipoprotein-lipase (LPL) in adipocytes and enhanced storage of liver-derived triglycerides [12], [13]. Despite the fact that non-pathogenic bacteria can promote proinflammatory changes in the intestine, the role of gut bacteria in diet-induced intestinal inflammation and the role of intestine inflammation in susceptibility to weight gain, obesity or insulin resistance have not to our knowledge been explored.

The current study tested the hypothesis that interactions exist between HF western diet and gut microbiota, which promote intestinal inflammation. To test this hypothesis we compared inflammation biomarkers in the intestine of conventionally raised (CONV) or GF mice given HF or LF diet. We also predicted that intestinal inflammation would precede or correlate with the development of obesity and/or insulin resistance and therefore examined how the timeframe of inflammatory changes in the intestine related to diet induced increases in body weight or fat mass, or insulin resistance. Levels of mRNA encoding TNF-α, a putative mediator of insulin resistance associated with obesity [15] and activation of an nuclear factor kappa-B (NF-κB) response element – enhanced Green Fluorescent Protein (EGFP) reporter transgene (NF-κB^EGFP^) were used as biomarkers of inflammation. Cellular sites of NF-κB^EGFP^ activation were also examined to define the cell-types in the intestine that exhibit HF diet-mediated inflammation. Our studies support a concept that HF diet in association with commensal gut microbiota promotes intestinal inflammation in multiple cell types. Onset of intestinal inflammation precedes diet-induced increases in body weight, fat mass and insulin resistance and degree of TNF-α induction strongly correlates with diet-induced increases in weight, adiposity, plasma glucose, and insulin.

## Methods

### Ethics Statement

All studies were approved by the Institutional Animal Care and Use Committee (IACUC) of the University of North Carolina at Chapel Hill (Approved protocol number: 07-229).

### Animals

Four week old CONV but specific pathogen free (SPF) C57BL/6 mice were purchased from Jackson Laboratories (Jackson labs, Bar Harbor, ME) and were subjected to dietary intervention in our SPF animal facility. GF C57BL/6 mice were bred in the UNC-CH gnotobiotic facility and maintained in GF conditions throughout the dietary interventions. NF-κB^EGFP^ knockin mice (C57BL/6) were described previously [16]. Briefly, this model contains a single copy of a transgene comprising NF-κB-response elements driving EGFP knocked into the hypoxanthine phosphoribosyltransferase genomic locus. These mice are maintained as hemizygotes and transgenics were identified by examining tail-clips for EGFP expression. Male CONV and GF NF-κB^EGFP^ mice were used for proposed studies.

### Diet intervention

CONV mice, CONV NF-κB^EGFP^ mice and GF mice were randomly selected to receive either HF diet (45% kcal from fat, D01060502, Research Diets, New Brunswick, NJ) or a LF diet (10% kcal from fat, D01060501, Research Diets, New Brunswick, NJ) for 2, 6, or 16 weeks (n = 4–8 per group). For experiments in GF mice, diets were subject to irradiation (50 kGy) (Neutron Products, MD). Pilot studies established that CONV mice given irradiated diet gained similar weight as on non-sterile diet.

### Body weight and body composition

Body weights were measured at two week intervals. Percentage body fat was assessed by performing Dual Energy x-ray Absorptiometry (DEXA) scan (PIXImus Mouse Densitometer, GE, Piscataway, NJ) at 2, 6, or 16 weeks after beginning the HF or LF diets. DEXA scans were obtained under isoflurane anesthesia to assess fat mass, percent fat and lean body mass.

### Sample Collection

Animals were euthanized with an overdose of sodium pentobarbital administered intraperitoneally. Cardiac puncture was used to obtain blood, which was placed on ice and centrifuged to collect plasma. Ileum and colon were dissected and carefully stripped of all adherent mesentery or fat. Corresponding segments of tissue from each animal were fixed in 10% formalin or 4% paraformaldehyde (PFA) for histology, or frozen for RNA extraction.

### Fasting plasma glucose and insulin measurement

Fasting plasma glucose was measured by a standard biochemical method (#439-90901, Wako Diagnostics, Richmond, VA) and fasting insulin by ELISA (#EZRMI-13K, rat/mouse insulin ELISA kit, Millipore, Billerica, MA). Homeostatic model assessment (HOMA) values were calculated as an estimate of insulin sensitivity, using the formula fasting plasma glucose (mmol/L)×insulin (µU/ml)/22.5. Higher values of HOMA indicate reduced insulin sensitivity.

### RNA extraction and Real-time PCR

Total RNA was isolated from ileum and colon using the TRIzol method (Invitrogen, Carlsbad, CA). cDNA was generated using AMV Reverse Transcriptase and Oligo(dT)_15_ primer in the presence of RNasin Ribonuclease Inhibitor. All reverse transcription reagents were obtained from Promega (Madison, WI). Real-time qPCR was performed on the Light Cycle (Rotor-Gene 3000, Corbett Life Science, San Francisco, CA). Primers and fluorescent reporter probes were obtained from Operon (Huntsville, AL). The sequences were as follows: TNF-α F: 5′-CTGTCTACTGAACTTCGGGGTGAT-3′, R: 5′-CATCAGTTCTATGGCCCAGACC-3′ and FAM-labeled probe: 5′-FAM-ATGAGAAGTTCCCAAATGGCCTCCCTC-TAMRA-3′). The results were normalized to housekeeping gene HMBS F: 5′-TGTGTTGCACGATCCTGAAAC-3′, R: 5′-CTCCTTCCAGGTGCCTCAGAA-3′ and 5′-FAM-TTCGCTGCATTGCTGAAAGGG-TAMRA-3′ probe. Cycle threshold (Ct) values for TNF-α and HMBS were converted to quantification values based on the standard curves. All individual values are expressed as a fold change compared to LF diet at the 2 week time point.

### Enhanced GFP (EGFP) imaging

NF-κB^EGFP^ mice given HF or LF diet were anesthetized and euthanized. The intestine was dissected and flushed with ice cold 1×PBS and immediately imaged using a charge-coupled device camera in a light-tight imaging box with a dual-filtered light source and emission filters specific for EGFP (LT-99D2 Illumatools; Lightools Research, Encinitas, CA). Intestines from HF/LF diet fed animals were also photographed using a Leica 16FA stereo dissecting microscope. Identical exposure times were used to capture images within each experiment.

### Cellular sites of NF-κB EGFP expression

GFP was visualized directly under confocal microscope on frozen section mounted in gel-mount. Immunofluorescence was performed on frozen sections cut from ileum and colon fixed in 4% PFA, cryoprotected in graded sucrose and embedded in O.C.T. (Tissue-Tek, Torrance, CA). Before the primary antibody incubation, frozen sections were pretreated with 0.05M Tris Triton (TT) epitope retrieval solution at room temperature (RT) for 30 minutes and blocked in 5% normal goat serum (NGS) diluted in TT buffer for 1 hr. The primary antibodies used are listed in Table 1. Primary antibodies were diluted in 5% NGS/TT buffer. The sections were incubated with primary antibodies overnight at 4°C in a humidity chamber. After washing 3 times in 0.05M Tris buffer, the sections were incubated with secondary antibodies (see Table 2) for 1 hr at RT. Nucleic acid staining was carried out by labeling with Bisbenzamide for 10 minutes. Following three washes with 0.05M Tris, coverslips were mounted in gel-mount (Electron Microscopy Sciences, Hatfield, PA). Pictures were taken by Leica SP2 Upright Laser Scanning Confocal microscope (Leica) and analyzed with Leica SP2 Upright Laser Scanning Confocal Imaging Software (Leica).

**Table 1:** Primary antibodies used in immunofluorescence.

Primary antibodies	Company	Cat. number	Dilution
GFP	Aves labs, Tigard, OR	F-1005	1∶500
F4/80	Abcam, Cambridge, MA	ab6640	1∶50
CD45R	Abcam, Cambridge, MA	ab64100	1∶200
CD3	Dako, Carpinteria, CA	A0452	1∶100
Neutrophil	Abcam, Cambridge, MA	ab2557	1∶50
CD11c	Abcam, Cambridge, MA	ab3385	1∶100
CD45	Biolegend, San Diego, CA	30-F11	1∶50
Substance P	Millipore, Billerica, MA	MAB356	1∶100
α-smooth muscle actin (SMA)	Dako, Carpinteria, CA	M0851	1∶200
PECAM-1	Santa Cruz Biotechnology, Santa Cruz, CA	sc-1506	1∶100

**Table 2:** Secondary antibodies used in immunofluorescence.

Secondary antibodies	Company	Cat. number	Dilution
Alexa Fluor 488 Goat anti chicken IgG	Invitrogen, San Diego, CA	A11039	1∶250
Alexa Fluor 568 Goat anti rabbit IgG	Invitrogen, San Diego, CA	A11036	1∶250
Alexa Fluor 568 Goat anti rat IgG	Invitrogen, San Diego, CA,	A11077	1∶250
Dylight Goat anti Hamster IgG	Jackson Immuno	127-505-099	1∶250

### Conventionalization of GF NF-κB ^EGFP^ with fecal slurries from mice given HF or LF diet

Fresh feces were obtained from CONV mice fed HF or LF diet for >16 weeks. Fecal slurries were made by suspending 1.2 g of HF/LF feces into 4 ml sterile 1×PBS (phosphate buffered saline). Whiskers and rectum of each mouse were swabbed with a cotton swab doused in fecal slurry. Colonized GF mice were then fed with sterilized regular chow diet and sterile water for 2 weeks before euthanasia.

### Statistical analysis

Data are expressed as mean ± standard error. Data in CONV or GF mice were compared for an effect of diet by One-Way ANOVA and post-hoc tests used for pair-wise comparisons (Tukey's test). Linear regression using Pearson's correlation coefficients was used to test for correlations between ileal TNF-α and body weight, fat mass, % fat, plasma glucose and insulin levels. All statistical analyses were performed using STATISTICA software version 9 (Statsoft inc. Tulsa, OK). A *P* value of less than 0.05 was considered statistically significant.

## Results

### HF diet increased body weight and percentage of body fat in CONV mice

**Figure 1:**
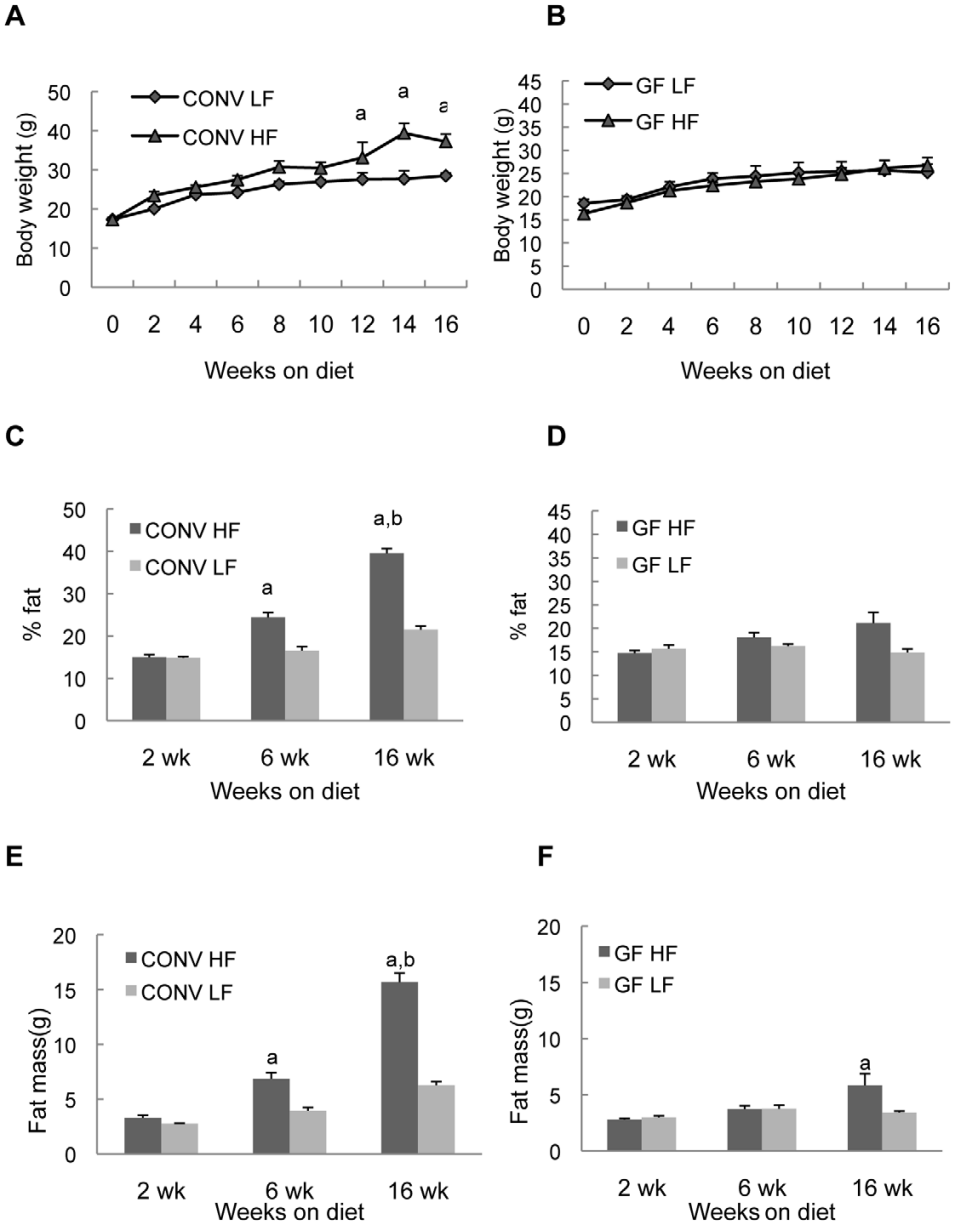
Body weight and fat mass in CONV or GF mice on HF/LF diet. Histograms show mean ± SE. a: *P*<0.05 vs LF group at the same time point; b: *P*<0.05 vs CONV HF at 2wk and 6wk.

CONV mice on HF and LF diets had similar body weights at the outset of the experiment (Figure 1). By week 12 and for the remainder of the experiment CONV mice fed HF diet showed statistically significant increases in body weight (*P*<0.01) relative to LF diet controls (Figure 1A). In contrast, GF mice fed HF vs LF diet did not differ in body weight at any time point (*P*>0.05 at all time points) and GF mice fed either HF or LF diet exhibited body weights over the 16 week period that were indistinguishable from CONV mice on LF diet (Figure 1A and B). CONV mice given HF and LF diet had similar percentage of body fat and fat mass at 2 weeks after onset of dietary intervention. By week 6, CONV mice on HF diet had significantly increased percent body fat and fat mass compared with the animals on LF diet and this increase was more dramatic by 16 weeks (*P*<0.001) (Figure 1C and E). Notably, GF mice fed HF diet showed no significant increase in percent body fat compared with GF mice fed LF diet at any time point (Figure 1D). GF animals did show small increase in fat mass but only after 16 weeks on HF diet, and the increase was much less than observed on CONV mice (Figure 1F). Together these data confirm prior findings [12], [13] that GF mice are resistant to diet induced weight gain or obesity.

### HF diet induced ileal TNF-α mRNA expression in CONV mice but not GF mice

**Figure 2:**
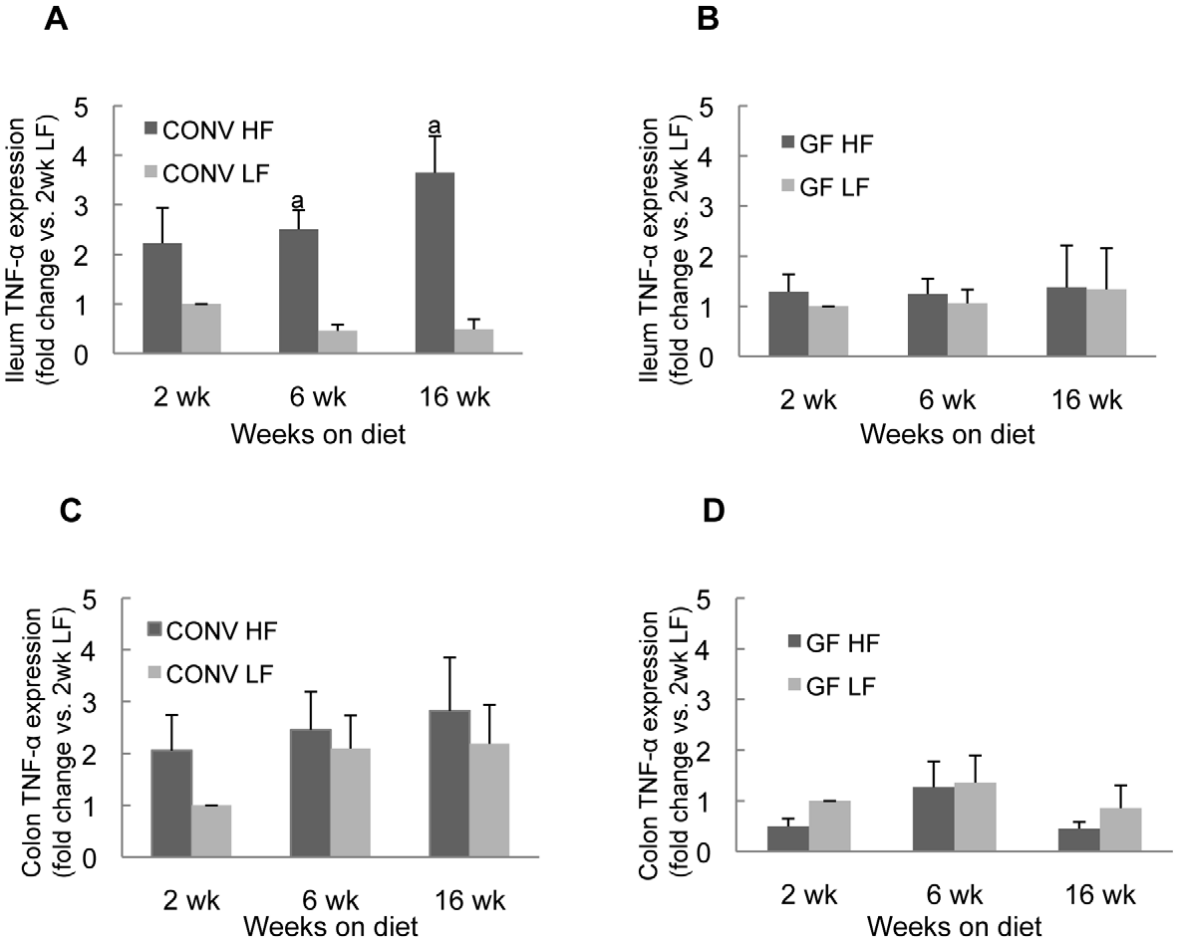
TNF-α mRNA levels in intestine of CONV or GF mice on HF/LF diet. Histograms show mean ± SE expressed as fold change vs the mean mRNA level in mice fed LF diet for 2 weeks. a: *P*<0.05 vs CONV LF diet group.

Real-time quantitative PCR for TNF-α was used as a sensitive biomarker of proinflammatory changes in the intestine. Ileum and colon were examined because we reasoned that HF diet was most likely to alter nutrient exposure in these segments and these regions have the highest densities of enteric bacteria in CONV mice. As shown in Figure 2, ileal TNF-α mRNA levels were significantly (*P*<0.01) increased in CONV mice at 6 and 16 weeks after onset of HF diet compared to CONV mice on LF diet (Figure 2A). In addition, after only two-weeks of HF diet, there was a trend towards a significant increase (2.22±0.72 vs 1.00) (*P* = 0.075) in levels of ileal TNF-α mRNA (Figure 2A). In contrast, in GF mice HF diet resulted in no significant change in ileal TNF-α mRNA at any time point (Figure 2B). HF diet did not significantly affect TNF-α mRNA levels in colon of either CONV or GF mice (Figures 2C and D). Thus ileal TNF-α mRNA, encoding a key proinflammatory cytokine, is induced by HF diet but only when natural enteric bacteria are present.

### Ileal TNF-α mRNA correlates with body weight and percent fat in CONV but not GF mice

Linear regression was used to test for potential association between ileal TNF-α mRNA and body weight or adiposity. In CONV mice, strong and significant correlations were observed between ileal TNF-α mRNA and percent fat at all time points whereas no significant correlations were observed in GF mice (Table 3). Body weight in CONV mice also significantly correlated with ileal TNF-α mRNA overall and at 6 and 16 weeks after onset of HF/LF diet and this relationship was not observed in GF mice. Correlations persist when we analyzed only mice on HF diet, suggesting that the levels of ileal TNF-α are significantly correlated with magnitude of HF-induced weight gain and obesity. (Table 4).

**Table 3:** Correlation coefficients between ileal TNF-α and body weight, adiposity in all CONV and GF mice on HF and LF diet at different time point.

	2 weeks		6 weeks		16 weeks	
	CONV	GF	CONV	GF	CONV	GF
Body weight	0.61	−0.06	0.72 ^a^	−0.54	0.75 ^a^	0.26
Fat mass	0.77 ^a^	−0.41	0.71 ^a^	−0.54	0.82 ^a^	0.32
% fat	0.82 ^a^	−0.36	0.67 ^a^	−0.46	0.87 ^a^	0.29

**Table 4:** Correlation coefficients between ileal TNF-α mRNA and body weight, adiposity in all CONV and GF mice on both diets and in CONV mice on HF diet only.

	All weeks		HF diet only
	CONV	GF	CONV
Body weight	0.51^a^	−0.18	0.55^a^
Fat mass	0.60^a^	−0.29	0.59^a^
% fat	0.63^a^	−0.31	0.61^a^

a: *P*<0.05.

### Ileal TNF-α mRNA and insulin resistance

**Figure 3:**
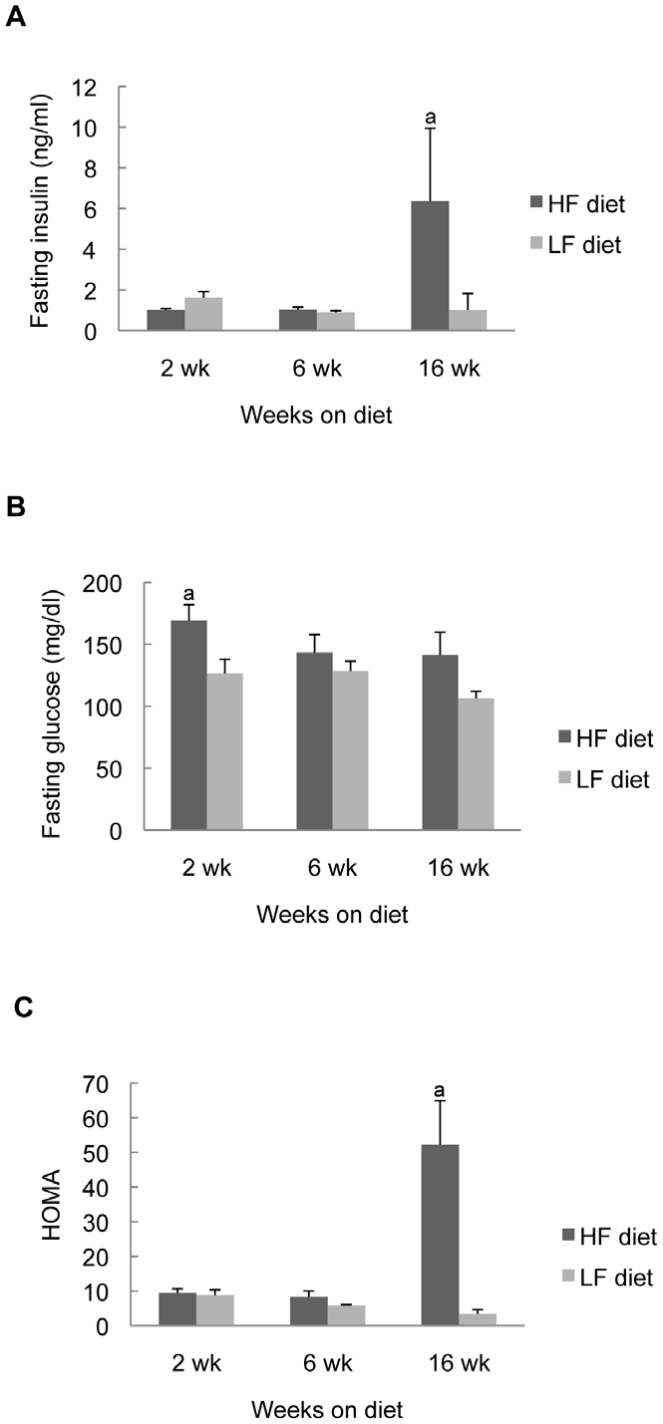
Metabolic measurements in CONV mice fed HF or LF diet. Data are expressed as mean ± SE; Note that food was withdrawn from mice 4 hours before plasma collection for insulin and glucose assays. a: *P*<0.05 vs LF group.

Since TNF-α has been linked to obesity associated insulin resistance, we assessed plasma glucose, insulin and HOMA in CONV mice fed HF or LF diet and tested for correlations with ileal TNF-α. As has been observed previously in CONV C57BL/6 mice, HF diet was associated with dramatic elevations in plasma insulin and HOMA but only after 16 weeks of diet (Figure 3A and 3C). Other than a small and transient increase in plasma glucose in mice fed HF diet for 2 weeks, plasma glucose did not differ significantly in CONV mice fed HF or LF diet (Figure 3B), indicating that the elevated insulin at 16 weeks was sufficient to maintain normal plasma glucose. Importantly in CONV mice ileal TNF-α showed strong and highly significant correlation with plasma insulin and plasma glucose (R values: insulin = 0.89, *P*<0.05; glucose = 0.93, *P*<0.05) at the 16-week time point (Table 5), and this was a particularly strong correlation. Together these data indicate that increased ileal TNF-α mRNA shows strong positive associations with weight gain, adiposity and subsequent development of insulin resistance induced by HF diet.

**Table 5:** Correlation coefficients between ileal TNF-α mRNA and plasma glucose and insulin levels in CONV mice fed HF and LF diets.

	All weeks	2 weeks	6 weeks	16 weeks
Glucose	0.25	0.07	0.32	0.93^a^
Insulin	0.53^a^	−1.29	0.40	0.89^a^

a: *P*<0.05.

### HF diet induced intestinal NF-κB ^EGFP^

**Figure 4:**
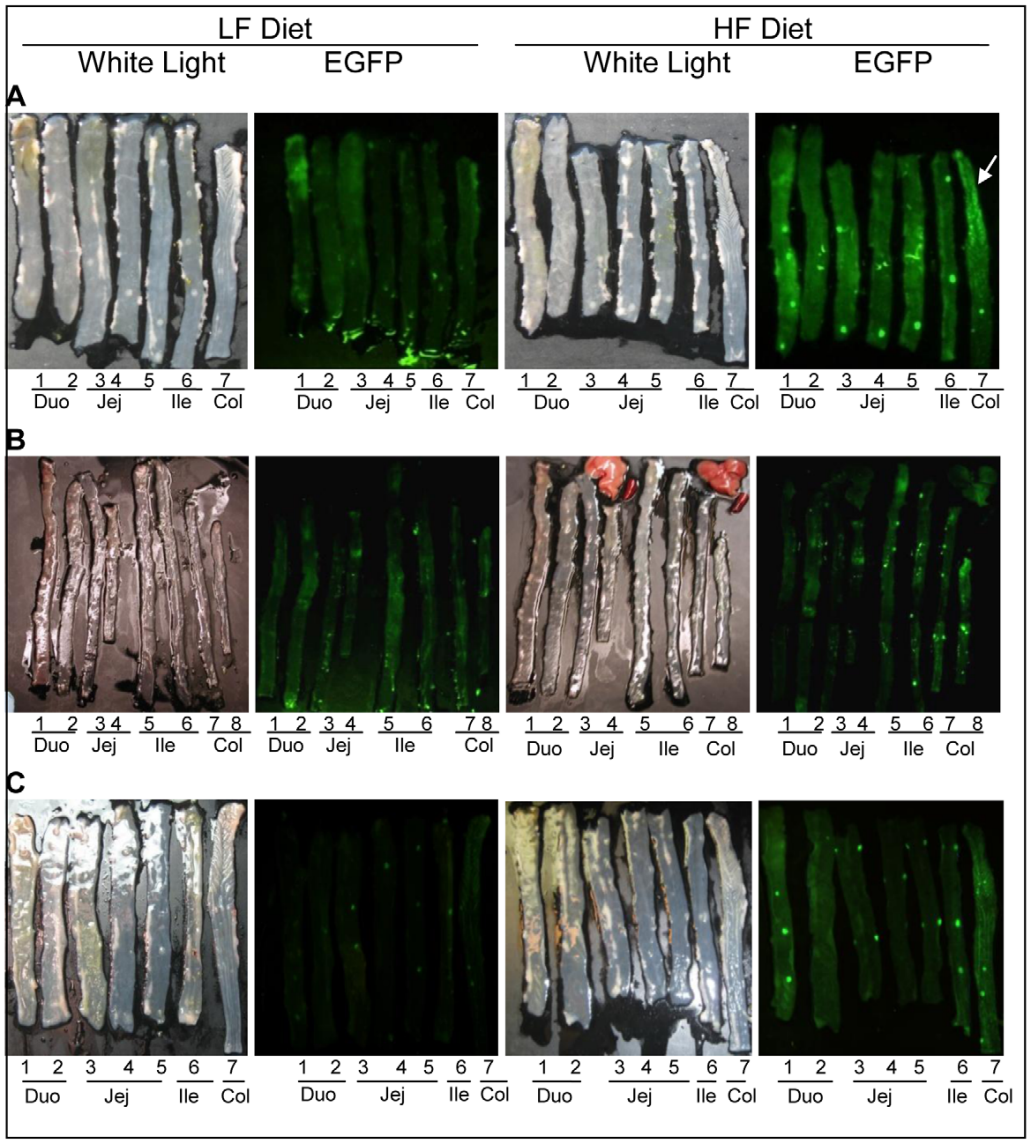
White light and EGFP imaging of intestine in CONV NF-κB^EGFP^ mice on HF/LF diet. White arrow: autofluorescence which is common in proximal colon. A: 2 weeks on HF/LF diet. B: 6 weeks on HF/LF diet. C: >16 weeks on HF/LF diet. Abbreviations: Duo: duodenum; Jej:Jejunum; Ile: ileum; Col:colon.

**Figure 5:**
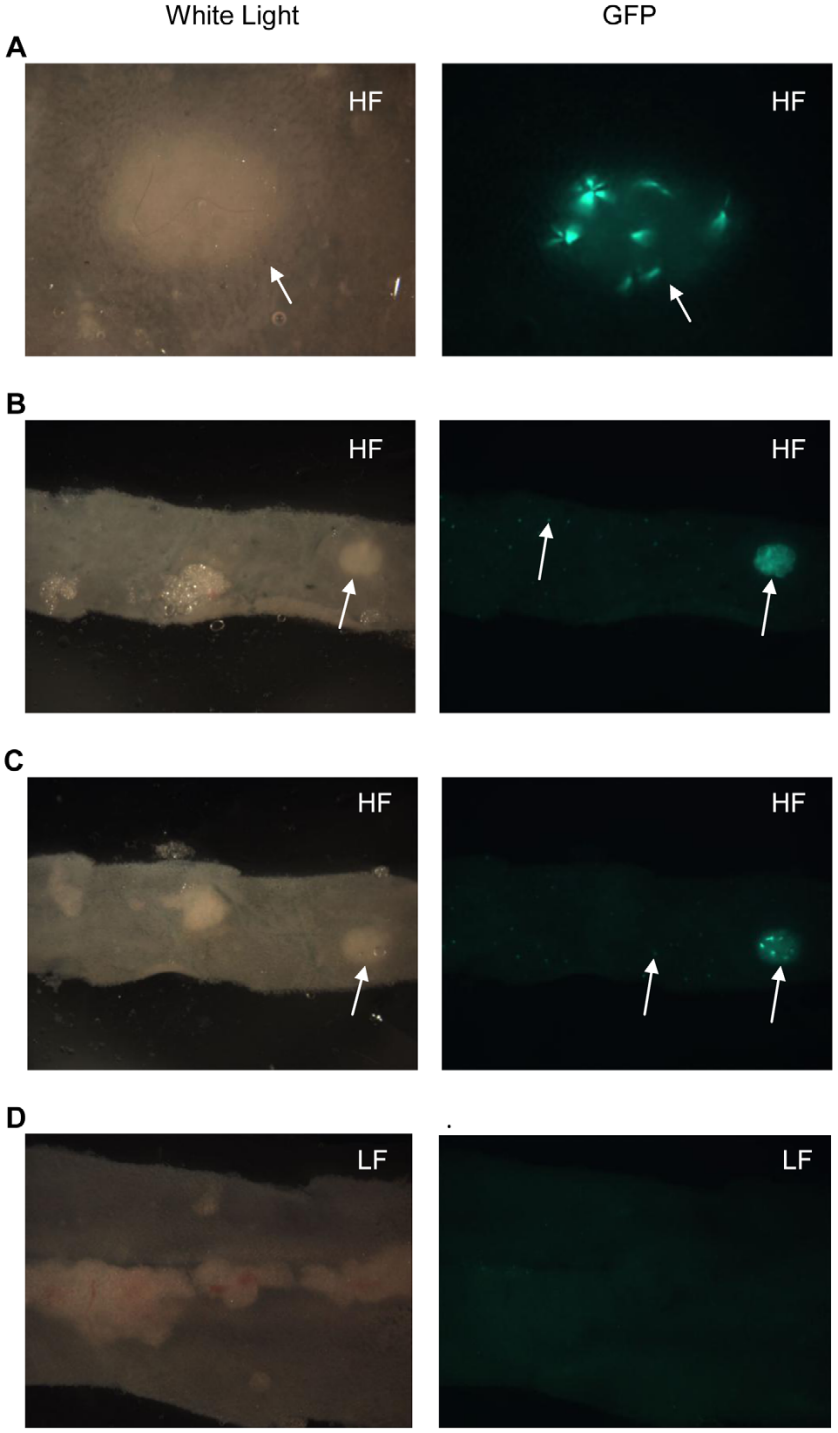
Imaging of small intestine in NF-κB^EGFP^ mice fed HF/LF diet for 16 weeks. A, B and C show representative images from 2 different mice given HF diet to illustrate large foci (Peyer's patches/lymphoid aggregates) and smaller foci (throughout the intestine) of NF-κB^EGFP^ expression (white arrows); D shows minimal NF-κB^EGFP^ activation in mice fed LF diet. A: large foci in jejunum (>7.11×magnification). B: large and small foci in ileum (7.11×magnification). C: large and small foci in ileum (7.11×magnification). D: large and small foci in distal ileum (>7.11×magnification).

**Figure 6:**
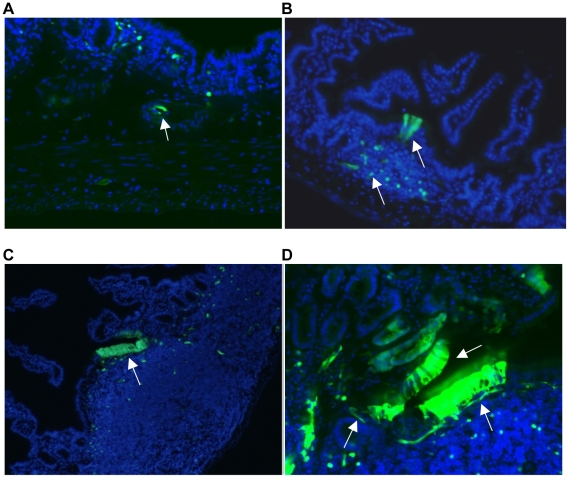
Representative regions of NF-κB^EGFP^ activation-epithelial cells over lymphoid aggregate, subepithelial cells, cells within blood vessel. A: NF-κB^EGFP^ activation in cells within blood vessel (10×). B: NF-κB^EGFP^ activation in epithelial cells over lymphoid aggregate (20×). C: NF-κB^EGFP^ activation in epithelial cells over lymphoid aggregate (10×). D: NF-κB^EGFP^ activation in epithelial cells over lymphoid aggregate and subepithelial cells (20×).

**Figure 7:**
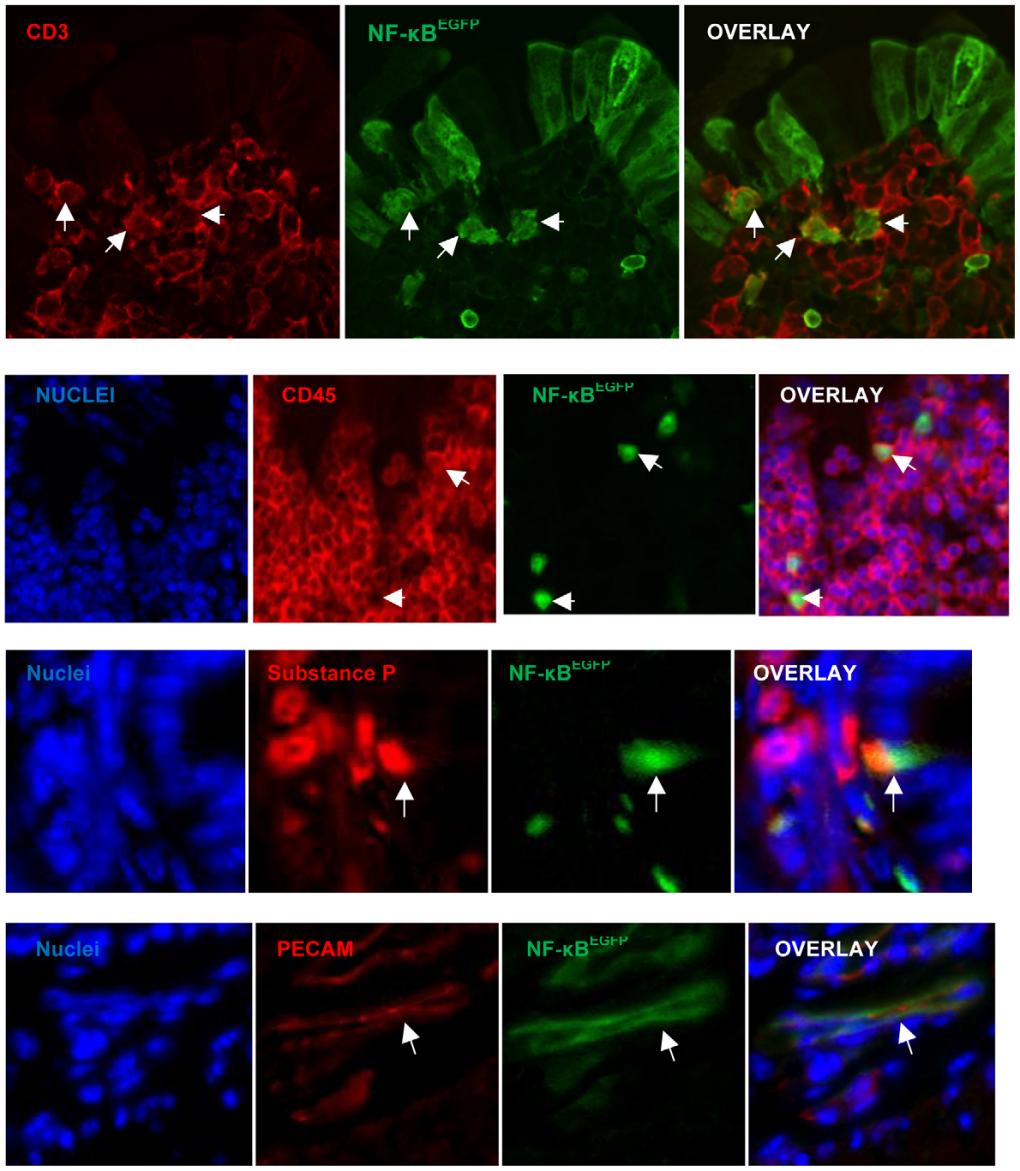
Immunostaining for antigenic markers (red) compared with NF-κB (green) and overlay showing colocalization (white arrows).

**Figure 8:**
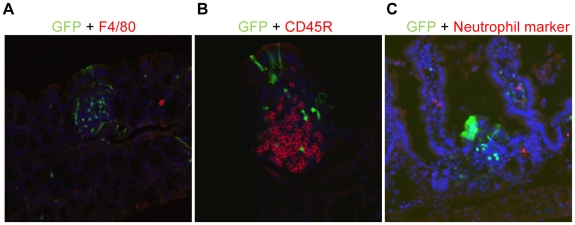
Immunostaining for antigenic markers (red) and GFP to show absence of co-localization in cells. (A) macrophages; (B)B cells; (C) neutrophils.

As an independent measure of intestinal inflammation induced by high fat diet, we fed CONV NF-κB^EGFP^ mice with HF or LF diet for 2, 6, or >16 weeks. At euthanasia, small intestine and colon were dissected and opened for fluorescence imaging. As shown in Figure 4, HF diet resulted in increased NF-κB^EGFP^ activation, throughout the entire small intestine and colon. This was evident at 2 weeks, suggesting that HF diet activated proinflammatory signaling in the intestine as early as 2 weeks after the diet intervention and this effect was maintained as long as HF diet continued (Figure 4). It should be noted that the informative and specific images in Figure 4 are the foci and bright patches of NF-κB^EGFP^ which are also readily evident at high power under the dissecting microscope. A diffuse signal (Figure 4) throughout the intestine of the HF animal at 2 weeks likely reflects background due to autofluorescence of traces of chow that are not completely removed upon flushing the intestine. This is particular evident in the proximal colon (indicated by the arrow) where it is difficult to completely remove traces of chow from mucosal folds. To confirm specific fluorescence signals, tissues were visualized at higher power under a dissecting microscope (Figure 5) and histologically (Figure 6). Figure 5 shows high power images viewed under a dissecting microscope which reveals large and small foci of inflammation in small intestine of NF-κB^EGFP^ mice fed HF diet for 16 weeks. Large foci represent histological analyses reveal that NF-κB^EGFP^ activation in Peyers Patches or lymphoid aggregates. The smaller foci of NF-κB^EGFP^ activation evident throughout the intestine of mice fed HF reflects NF-κB^EGFP^ activation in epithelial cells and multiple types of sub-epithelial cell (Figures 6, 7 and 8).

### Fecal slurries from mice fed HF diet are sufficient to activate NF-κB^EGFP^ in GF NF-κB^EGFP^ mice

**Figure 9:**
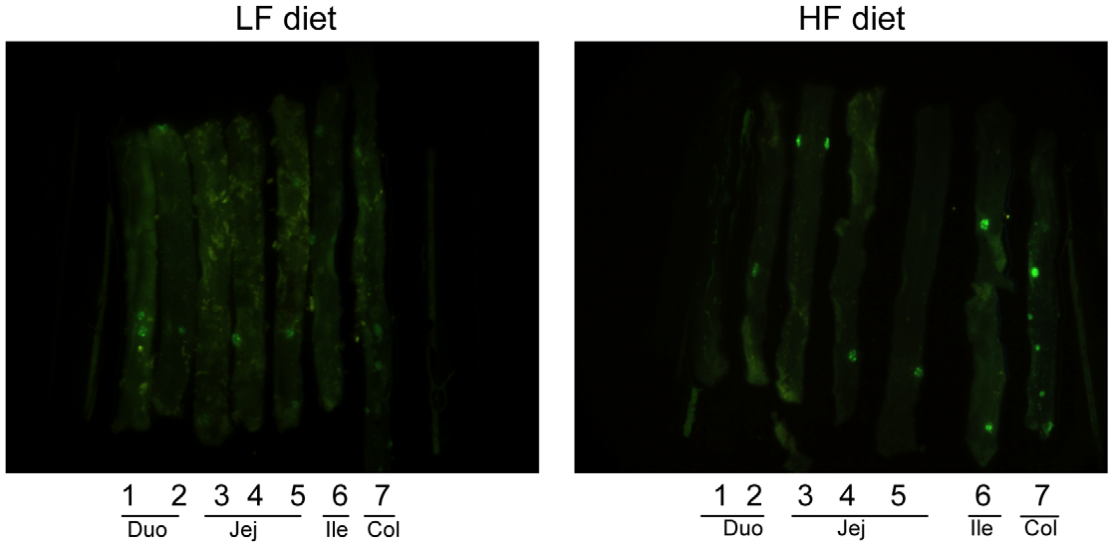
NF-κB^EGFP^ in intestinal tissue of GF mice treated with HF or LF fecal slurry. Fecal slurry from mice fed HF diet induces NF-κB^EGFP^ expression to a greater extent than fecal slurries from mice fed LF diet (Image representative of observation in 3 pairs of mice) in intestine tissues of GF NF-κB^EGFP^ mice. Abbreviations: Duo: duodenum; Jej:Jejunum; Ile: ileum; Col:colon.

We colonized GF NF-κB^EGFP^ mice with fecal slurries from CONV mice fed HF or LF diet, then fed both groups standard chow and examined NF-κB^EGFP^ activation two weeks later. Imaging showed enhanced NF-κB^EGFP^ activation in mice colonized with HF fecal slurry compared with those colonized by LF fecal slurry (Figure 9).

### Cellular sites of NF-κB^EGFP^ activation in CONV mice fed HF diet

To identify the cells with activated NF-κB^EGFP^, we localized NF-κB^EGFP^ fluorescence in sections of ileum. NF-κB^EGFP^ was observed in multiple cell types including intestinal epithelial cells, sub-epithelial cells, some, but not all cells within sub-epithelial immune aggregates and cells within blood vessels (Figure 6). We note that the patterns of NF-κB^EGFP^ induction are striking with focal patches of epithelial cells showing NF-κB^EGFP^ induction. This may reflect localized effects of diet∶bacteria interactions to activate NF-κB. The GFP is present throughout the epithelial cells including the brush border. This pattern is similar to that reported in LPS treated NF-κB^EGFP^ mice [16]. Since GFP accumulates within the cell due to activation of upstream NF-κB regulatory elements its subcellular localization is not functionally relevant and reflects diffusion of GFP throughout the intestinal epithelial cell. To better define the cellular sites of NF-κB^EGFP^ induction by HF diet in other cell types, co-immunofluorescence (IF) was performed for a number of cellular antigens and EGFP. IF for GFP confirmed expression in intestinal epithelial cells, subsets of CD3+ and CD45+ immune cells, rare enteroendocrine cells expressing substance P and PECAM positive endothelial cells (Figure 7). Thus HF diet induces inflammatory changes in multiple cell types. Interestingly, as shown in Figure 8, we could find no co-expression of GFP and antigenic markers of mucosal macrophages (F4/80), neutrophils, B cells (CD45R) (Figure 8), dendritic cells (CD11c) and smooth muscle actin positive myofibroblasts or smooth muscle cells (did not shown).

## Discussion

Inflammation associated with obesity is considered central to the development of complications such as insulin resistance. Although the intestine is the first organ exposed to ingested nutrient has an intrinsic resident immune system, surprisingly little attention has focused on the potential effect of diet on intestinal inflammation or its association with weight gain or complications such as insulin resistance. Normal commensal enteric bacteria are known to trigger a low grade inflammatory response in the intestine and are also increasingly linked to DIO [12], [17], [18]. However, the potential role of commensal microbiota in diet-induced intestinal inflammation has not been widely studied. The current study tested the hypothesis that HF diet interacts with intestinal bacteria to induce intestinal inflammation. In support of this hypothesis, our study revealed that HF diet induces two inflammatory biomarkers in the intestine, TNF-α mRNA and NF-κB activation. Furthermore, the presence of enteric bacteria is required for HF diet to induce TNF-α and NF-κB since GF mice given HF diet did not exhibit up-regulation of these markers. Strong correlation between ileal TNF-α mRNA, weight gain, adiposity, plasma insulin and glucose in CONV mice fed HF diet, and the fact that induction of intestinal inflammation biomarkers preceded diet-induced weight gain and adiposity suggests that the role of intestinal TNF-α and intestinal inflammation in DIO and complications should be further explored.

### Bacteria and HF diet interaction leads to intestinal inflammation and obesity

Our finding that GF mice are resistant to DIO, weight gain and adiposity is consistent with recent findings of Backhed [12], [13]. However, in contrast to Backhed, we did not observe increased body weight, % fat or fat mass in age-matched CONV mice versus GF mice at baseline. In GF mice, HF diet did lead to a small increase in fat mass by 16 weeks, but this was much less dramatic than in CONV mice, which also showed earlier HF-diet induced increases in fat mass than GF mice. These data underscore that an interaction between bacteria and HF diet produces earlier and larger changes in body weight and adiposity than either of the factors individually.

Our finding that CONV but not GF mice showed induction of ileal TNF-α after HF diet demonstrates that an interaction between enteric microbiota and HF diet must occur to elicit intestinal inflammation. A number of studies indicate that HF diet alters the composition of bacteria, favoring high levels luminal Firmicutes and Proteobacteria and lower levels of Bacteroidetes [14], [19]. Other studies have demonstrated that HF diet increases the proportion of lipopolysaccharide (LPS) –containing gut bacteria and increases intestinal permeability leading to elevated circulating LPS concentrations [17]. Such diet induced changes in intestinal microbiota and intestinal permeability may contribute to the intestinal inflammation observed in CONV mice fed HF diet. Findings that fecal slurries from animals fed HF diet were sufficient to enhance activation of the NF-κB^EGFP^ reporter in animals fed normal chow support a concept that alterations in gut microbiota due to HF diet are sufficient to elicit intestinal inflammation even in animals consuming normal chow. Similarly a recent study demonstrated that HF diet altered the composition of luminal bacteria in mice genetically engineered to be resistant to obesity [19]. Thus, diet-associated alterations in intestinal microbiota can precede obesity.

### HF diet-induced inflammation in CONV mice precedes weight gain and adiposity

In CONV mice fed HF diet, increases in TNF-α mRNA and NF-κB^EGFP^ activation preceded HF diet induced weight gain and preceded or occurred coincidentally with the earliest detectable increases in fat mass. Thus levels of intestinal TNF-α mRNA, using highly sensitive real-time PCR or the NF-κB^EGFP^ reporter represent a useful biomarker of early proinflammatory effects of HF diet on intestine. It is of interest that the HF diet associated increase in TNF-α occurred in ileum and not in colon, suggesting that future evaluation of effects of HF diet specifically on small bowel microbiota will be of interest. The induction of these proinflammatory biomarkers prior to the onset of significant weight gain (at 8 weeks) and measurable increases in adiposity (6 weeks), contrast with adipose inflammation, which typically occurs after onset of DIO. This supports a concept that early proinflammatory effects of HF diet∶bacteria interactions in the intestine could serve as a trigger for subsequent inflammation in other organs or systemic inflammation.

Evaluation of cellular sites of intestinal inflammation induced by HF diet in CONV mice based on NF-κB^EGFP^ reporter activation indicate involvement of multiple cell types including epithelial cells, intramucosal CD45 positive leukocytes, and CD3 positive T cells, and endothelial cells. Proinflammatory molecules released by these cells are ideally placed to enter the hepatic circulation and could impact on development of systemic inflammation or insulin resistance. It is of interest that epithelial sites of NF-κB-EGFP activation by HF diet were not uniform but focal. This suggests that local adherence of bacteria or production of proinflammatory signaling may contribute to HF diet induced intestinal inflammation. A small number of enteroendocrine cells also showed NF-κB^EGFP^ reporter activation. This is intriguing because recent studies indicate that prebiotics, which alter gut microbiota and intestinal permeability, can impact on release of gut hormones such as glucagon-like peptide 2 (GLP-2) [20]. Our findings that HF-diet in conjunction with the presence of bacteria induces inflammatory signaling in enteroendocrine cells suggests that modulation of enteroendocrine cell function may contribute to the impact of diet∶microbial interactions in mediating obesity.

### Induction of TNF-α in small intestine precedes and correlates with the development of insulin resistance and obesity

It is well known that overproduction of TNF-α by macrophages in adipose tissue is an important feature of obesity which is thought to contribute significantly to the development of insulin resistance [21], [22]. Numerous studies have demonstrated that TNF-α limits insulin receptor signaling and promotes insulin resistance [15], [23]. Additionally, mice with deletion of the TNF-α gene, TNF-α converting enzyme or receptors for TNF-α are partially resistant to diet induced obesity and insulin resistance [24], [25], [26], [27]. The observation here that increases in intestinal TNF-α precede but strongly correlate with weight gain, body fat, and subsequent development of insulin resistance, supports a potential role of intestinal derived TNF-α in the development of DIO and associated insulin resistance. CONV mice became overtly insulin resistant as manifest by increased HOMA by 16 weeks after onset of HF diet. Consistent with prior findings [12], [13], GF mice did not develop insulin resistance as indicated by the lack of significant difference in HOMA at 16 weeks in GF fed HF = 8.5±2.8 vs GF fed LF = 4.7±0.7, *P*>0.05. These values are much lower (*P*<0.05) than the HOMA in CONV mice fed HF diet for 16 weeks (52.2±12.7).

TNF-α activates NF-κB and other inflammatory pathways involved in the etiology of insulin resistance and Type 2 diabetes [28], [29]. Blocking these pathways can alleviate some of the symptoms of insulin resistance [15], [30]. It has been reported that blocking NF-κB in macrophages or liver attenuated inflammatory gene expression and insulin resistance on HF diet [31], c-Jun amino-terminal kinase 1 (JNK1) knockout mice do not become insulin resistant [32] while blocking JNK improves insulin sensitivity [33]. These results indicate that these inflammatory pathways (e.g. IKK, JAK/STAT) may serve as effective targets to prevent or alleviate the onset of insulin resistance. To date, adipose-derived macrophages or hepatocytes and hepatic Kupffer cells have been considered key targets of these beneficial effects of anti-inflammatory therapy. Our findings that HF diet ∶ bacteria interactions promote proinflammatory signaling in multiple cell types in the intestine, although surprisingly not gut macrophages, suggest that future evaluation of the effects of anti-inflammatory therapies on HF diet induced gut inflammation and subsequent weight gain and insulin resistance could be of considerable interest.

In conclusion, our study demonstrates that HF diet and enteric bacteria interact to promote inflammatory changes in the intestine, prior to the development of weight gain, adiposity, and insulin resistance. The magnitude of induction of the intestinal inflammatory biomarker, TNF-α, strongly correlates with HF-diet induced weight gain, adiposity and insulin resistance in CONV animals with intact commensal microbiota. These findings suggest that diet-induced intestinal inflammation may serve as a useful biomarker for risk assessment of diet-induced obesity and complications, and that strategies to limit this effect of HF diet may be beneficial in combating risk of obesity and insulin resistance.

## References

[1] Lee DE, Kehlenbrink S, Lee H, Hawkins M, Yudkin JS (2009). Getting the message across: mechanisms of physiological cross talk by adipose tissue.. Am J Physiol Endocrinol Metab.

[2] Bgreenberg AS, Obin MS (2006). Obesity and the role of adipose tissue in inflammation and metabolism.. J Clin Nutr.

[3] Trayhurn P, Bing C, Wood S (2006). Adipose tissue and adipokines-energy regulation from the human perspective.. J Nutr.

[4] Kern PA, Ranganathan S, Li C, Wood L, Ranganathan G (2001). Adipose tissue tumor necrosis factor and interleukin-6 expression in human obesity and insulin resistance.. Am J Physiol Endocrinol Metab.

[5] Syrenicz A, Garanty-Bogacka B, Syrenicz M, Gebala A, Walczak M (2006). Low-grade systemic inflammation and the risk of type 2 diabetes in obese children and adolescents.. Neuro Endocrinol Lett.

[6] Panagiotakos DB, Pitsavos C, Yannakoulia M, Chrysohoou C, Stefanadis C (2005). The implication of obesity and central fat on markers of chronic inflammation: The ATTICA study.. Atherosclerosis.

[7] Visser M, Bouter LM, McQuillan GM, Wener MH, Harris TB (2001). Low-grade systemic inflammation in overweight children.. Pediatrics.

[8] Dandona P, Weinstock R, Thusu K, Abdel-Rahman E, Aljada A (1998). Tumor necrosis factor-alpha in sera of obese patients: fall with weight loss.. J Clin Endocrinol Metab.

[9] Brake DK, Smith EO, Mersmann H, Smith CW, Robker RL (2006). ICAM-1 expression in adipose tissue: effects of diet-induced obesity in mice.. Am J Physiol Cell Physiol.

[10] Xu H, Barnes GT, Yang Q, Tan G, Yang D (2003). Chronic inflammation in fat plays a crucial role in the development of obesity-related insulin resistance.. J Clin Invest.

[11] Weisberg SP, McCann D, Desai M, Rosenbaum M, Leibel RL (2003). Obesity is associated with macrophage accumulation in adipose tissue.. J Clin Invest.

[12] Bäckhed F, Ding H, Wang T, Hooper LV, Koh GY (2004). The gut microbiota as an environmental factor that regulates fat storage.. Proc Natl Acad Sci.

[13] Bäckhed F, Manchester JK, Semenkovich CF, Gordon JI (2007). Mechanisms underlying the resistance to diet-induced obesity in germ-free mice.. Proc Natl Acad Sci.

[14] Turnbaugh PJ, Ley RE, Mahowald MA, Magrini V, Mardis ER (2006). An obesity-associated gut microbiome with increased capacity for energy harvest.. Nature.

[15] Moller DE (2000). Potential role of TNF-α in the pathogenesis of insulin resistance and type 2 diabetes.. Trends Endocrinol Metab.

[16] Magness ST, Jijon H, Van Houten Fisher N, Sharpless NE (2004). In vivo pattern of lipopolysaccharide and anti-CD3-induced NF-κB activation using a novel gene-targeted enhanced GFP reporter gene mouse.. J Immunol.

[17] Cani PD, Amar J, Iglesias MA (2007). Metabolic endotoxemia initiates obesity and insulin resistance.. Diabetes.

[18] Cani PD, Delzenne NM, Amar J, Burcelin R (2008). Role of gut microflora in the development of obesity and insulin resistance following high-fat diet feeding.. Pathologie Biologie.

[19] Hildebrandt MA, Hoffmann C, Sherrill-mix SA, Keilbaugh SA, Hamady M (2009). High-fat diet determines the composition of the murine gut microbiome independently of obesity.. Gastroenterology.

[20] Cani PD, Possemiers S, Van de Wiele T, Guiot Y, Everard A (2009). Changes in gut microbiota control inflammation in obese mice through a mechanism involving GLP-2-driven improvement of gut permeability.. Gut.

[21] De Taeye BM, Novitskaya T, McGuinness OP, Gleaves L, Medda M (2007). Macrophage TNF-alpha contributes to insulin resistance and hepatic steatosis in diet-induced obesity.. Am J Physiol Endocrinol Metab.

[22] Lumeng CN, Bodzin JL, Saltiel AR (2007). Obesity induces a phenotypic switch in adipose tissue macrophage polarization.. J Clin Invest.

[23] Aguirre V, Werner ED, Giraud J, Lee YH, Shoelson SE (2002). Phosphorylation of Ser307 in insulin receptor substrate-1 blocks interactions with the insulin receptor and inhibits insulin action.. J Biol Chem.

[24] Uysal KT, Wiesbrock SM, Marino MW, Hotamisligil GS (1997). Protection from obesity-induced insulin resistance in mice lacking TNF-α function.. Nature.

[25] Serino A, Menghini R, Fiotentino L, Amoruso R, Mauriello A (2007). Mice heterozygous for Tumornecrosis factor-α converting enzyme are protected from obesity-induced insulin resistance and diabetes.. Diabetes.

[26] Pamir N, McMillen TS, Kaiyala KJ, Schwartz MW, LeBoeuf RC (2009). Receptors for tumor necrosis factor-alpha play a protective role against obesity and alter adipose tissue macrophage status.. Endocrinology.

[27] Romanatto T, Roman EA, Arruda AP, Denis RG, Solon C (2009). Deletion of tumor necrosis factor-alpha receptor 1 (TNFR1) protects against diet-induced obesity by means of increased thermogenesis.. J Biol Chem.

[28] Barnes PJ, Karin M (1997). Nuclear factor κB: a pivotal transcription factor in chronic inflammatory diseases.. N Engl J Med.

[29] Yuan M (2001). Reversal of Obesity and diet-induced insulin resistance with salicylates or targeted disruption of IKKbeta.. Science.

[30] Ueki K, Kondo T, Tseng YH, Kahn CR (2004). Central role of suppressors of cytokine signaling proteins in hepatic steatosis, insulin resistance, and the metabolic syndrome in the mouse.. Proc Natl Acad Sci.

[31] Arkan MC, Hevener AL, Greten FR, Maeda S, Li ZW (2005). IKK-beta links inflammation to obesity-induced insulin resistance.. Nat Med.

[32] Hirosumi J, Tuncman G, Chang L, Görgün CZ, Uysal KT (2002). A central role for JNK in obesity and insulin resistance.. Nature.

[33] Kaneto H, Nakatani Y, Miyatsuka T, Kawamori D, Matsuoka TA (2004). Possible novel therapy for diabetes with cell-permeable JNK-inhibitory peptide.. Nat Med.

